# Detection of Extracellular Vesicle-Derived RNA as Potential Prostate Cancer Biomarkers: Role of Cancer-type SLCO1B3 and ABCC3

**DOI:** 10.7150/jca.90836

**Published:** 2024-01-01

**Authors:** Kristi Y. Lee, Erica L. Beatson, Martina A. Knechel, Elijah R. Sommer, Giulia C. Napoli, Emily N. Risdon, Andres F. Leon, Roger D. Depaz, Jonathan D. Strope, Douglas K. Price, Cindy H. Chau, William D. Figg

**Affiliations:** 1 Molecular Pharmacology Section, Genitourinary Malignancies Branch, Center for Cancer Research, National Cancer Institute, National Institutes of Health, Bethesda, MD, USA.; 2 Clinical Pharmacology Program, Center for Cancer Research, National Cancer Institute, National Institutes of Health, Bethesda, MD, USA.

**Keywords:** organic anion transporter family 1B3, SLCO1B3, ABCC3, prostate cancer, extracellular vesicle, cancer biomarker

## Abstract

Extracellular vesicles (EVs) provide a minimally invasive liquid biopsy source of tumor-specific markers for patients who have already undergone prostatectomies. Our laboratory has previously demonstrated enrichment of the cancer-type solute carrier organic anion transporter family 1B3 (*ct-SLCO1B3*) and the ATP Binding Cassette Subfamily Member C (*ABCC3*) in castration-resistant cell lines (CRPC). However, their expression in EVs has yet to be explored. Our study demonstrated that ct-SLCO1B3 and ABCC3 are highly detectable in CRPC cell line-derived EVs. We also showed that *ct-SLCO1B3* and* ABCC3* were detectable in a CRPC xenograft mouse model, both intratumorally and in plasma-derived EVs. Our results provide evidence for EV-contained *ct-SLCO1B3* and *ABCC3* as novel, EV-based tumor markers for prostate cancer progression.

## Introduction

Prostate cancer (PCa) is the most commonly diagnosed cancer among American males, accounting for an estimated 34,700 deaths in the United States in 2023 1. Patients who undergo androgen deprivation therapy (ADT) for advanced disease inevitably progress to castration-resistant prostate cancer (CRPC) 2, characterized by tumor progression despite castrate levels of serum testosterone (<50 ng/dL) 3. Present monitoring of CRPC emergence relies heavily on prostate-specific antigen (PSA); however, PSA is prostate-specific rather than PCa-specific, complicating its utility as a marker of disease progression 4. While numerous multifaceted liquid-biopsy diagnostic panels aimed at refining the staging available for males with PCa have been developed, such as the prostate health index (PHI) and prostate 4Kscore test 5, further identification and incorporation of additional biomarkers into clinical decision-making may enable improved tracking of disease progression.

Extracellular vesicles (EVs), such as exosomes and microvesicles, are spherical, membrane-bound particles containing proteins, metabolites, or nucleic acids that offer a snapshot of the cells they derive from 6, 7. While EVs are produced by healthy cells for normal physiological processes, they are also secreted by tumor cells, affecting the invasive potentials to both proximal and distant tissues 8. As such, EVs represent a novel and minimally invasive source of tumor-specific markers, as they are easily collected from a variety of biofluids (e.g. seminal fluid, plasma, urine, etc.) and contain a stable population of different biomolecules (e.g. RNA, DNA, protein, and lipids, etc.) 9, 10. Liquid-based biopsy methods offer a more comprehensive picture of genetic heterogeneity in both primary tumors and metastases compared to tissue biopsies 11.

EVs are a promising source of diagnostic, prognostic, and predictive biomarkers for prostate cancer. While the lipidomic and metabolic profile of prostate cancer-derived EVs has been examined 12, 13, EV protein and RNA are the most commonly explored avenues for potential biomarkers 14-16. For example, the commercially available ExoDx™ Prostate test uses the expression of RNA transcripts PCA3, ERG, and SPDEF in urinary exosomes to predict the likelihood of clinically significant prostate cancer (Gleason Score ≥7) in patients on active surveillance 17. This test outperformed multivariate risk calculators from both the Prostate Cancer Prevention Trial and European Randomized Prostate Cancer Study of Screening for Prostate Cancer in a homogenous risk group of pre-biopsy patients with no history of PCa 18. Beyond early detection, various markers from prostate cancer-derived EVs correlate with tumor progression and metastasis, disease severity, and survival 14, 19, 20. Many of those identified markers include microRNAs, some of which have been clinically validated as reliable indicators of CRPC 21, 22. EV-derived biomarkers have also been used to predict treatment response to ADT 23, 24.

The organic anion transporting polypeptide 1B3 (OATP1B3, encoded by *SLCO1B3*) transporter modulates intratumoral androgen concentration uptake that promotes CRPC progression. The liver-type OATP1B3 (lt-OATP1B3) is an androgen transporter, and polymorphic variations in the *SLCO1B3* gene are associated with poor overall survival from diagnosis, poor progression-free survival on androgen deprivation therapy biochemical recurrence and prostate cancer-specific mortality 25-29. Despite being found in many cancerous tissues, very little is known about the function of the cancer-type OATP1B3 (ct-OATP1B3), a variant isoform of lt-OATP1B3 30. While the well-characterized lt-OATP1B3 is found on healthy hepatocytes, ct-OATP1B3 primarily localizes to the cytosol in cancer cells and exhibits modest transport activity 31. Thus, the functional significance of ct-OATP1B3 in prostate cancer remains to be elucidated. Our laboratory has previously shown that while *ct-SLCO1B3* is only minimally expressed in the androgen receptor (AR)-positive PCa cell lines (e.g. LNCaP and 22Rv1), the transcript is highly expressed in the CRPC (AR null) cell lines, DU-145 and PC3 27. In addition to *ct-SLCO1B3,* the ATP binding cassette (*ABCC3*) transporter was identified as the most highly differentially expressed gene among CRPC cell lines. *ABCC3* expression has been implicated in multidrug resistance 32. However, there are limited studies on the role of both transporters in prostate cancer. Evidence of *ct-SLCO1B* and* ABCC3 e*xpression in CRPC cell lines suggests their potential utility as potential markers for disease progression. While *ct-SLCO1B3* has been previously investigated as an EV-derived tumor marker in both squamous cell carcinoma and colorectal cancer 6, 7, its role as a potential liquid biopsy EV-derived marker in prostate cancer has yet to be explored.

This study investigated whether ct-SLCO1B3 and ABCC3 can be detected in prostate cancer-derived EVs. Our results provide experimental evidence that both genes are limited to CRPC-derived EVs and are readily detectable in preclinical plasma samples, highlighting their potential as EV-based liquid biopsy tumor markers for CRPC disease progression.

## Materials and Methods

### Cell Culture

All prostate cancer cell lines [LNCaP (RRID: CVCL_0395), 22Rv1 (RRID: CVCL_1045), PC3 (RRID: CVCL_0035), and DU-145 (RRID: CVCL_0105)] were purchased from American Type Culture Collection (ATCC, Manassas, VA). Unless otherwise specified, cell culture reagents were obtained from ThermoFisher Scientific (Waltham, MA). LNCaP and 22Rv1 cells were maintained in phenol red-free RPMI 1640 medium supplemented with 10% fetal bovine serum (R and D Systems, Minneapolis, MN) and 1% Penicillin-Streptomycin (P/S). PC3 cells were maintained in F12K Nutrient Mixture, and DU-145 cells were maintained in Dulbecco's Modified Eagle Medium, supplemented with 10% FBS and 1% P/S. Cells were incubated at 37°C in an atmosphere containing 5% CO_2_ and 95% humidity. All cell lines used in this study were authenticated by ATCC and routinely tested for mycoplasma contamination.

### EV Isolation and Western Blot Analysis

Human prostate cancer cell lines were grown to confluence, washed with Dulbecco's phosphate-buffered saline, and cultivated for two days in serum-free medium. EVs from 25 mL pre-filtered (0.8 µM Millex-AA filter, Millipore, Billerica, MA) culture medium were isolated using the exoEasy Maxi Kit protocol (QIAGEN, Germantown, MD). Eluates containing intact vesicles were concentrated using the 100K Amicon Ultra-0.5 mL Centrifugal Filter (Millipore). To obtain whole cell lysates, cells were lysed with 1 mL RIPA buffer (Sigma-Aldrich, St. Louis, MO) + 1% protease inhibitor cocktail (Nacalai USA, San Diego, CA). After 30 minutes of incubation on ice, cell lysates were vortexed briefly, incubated an additional 30 minutes on ice, and centrifuged at 7,500 x g for 10 minutes. Supernatants were collected, and total protein concentration was determined using the Pierce™ BCA Protein Assay Kit (Thermo Scientific™, Waltham, MA), according to the manufacturer's protocol. Equivalent amounts of lysate protein and concentrated EV sample were heated to 95°C in Laemmli sample buffer (Bio-Rad, Hercules, CA) + 10% β-mercaptoethanol. Proteins were separated through SDS-PAGE using the 4-20% Mini-PROTEAN^®^ TGX™ gel (Bio-Rad) and Tris/Glycine/SDS running buffer (Bio-Rad). Separated proteins were transferred to a nitrocellulose membrane using the Trans-Blot Turbo Transfer System (Bio-Rad). Blocking was performed for 1 hour at room temperature with 5% nonfat milk in Tris-buffered saline + 0.1% Tween (TBS-T). The membrane was incubated overnight at 4°C with the following primary antibodies: mouse anti-CD81 IgG1 (diluted 1:1000 in TBS-T, 10630D, Invitrogen, Waltham, MA) and rabbit anti-GOLGA2/GM130 IgG (diluted 1:2000 in TBS-T, 11308-1-AP, Proteintech, Rosemont, IL). The membrane was then incubated for 1 hour at room temperature with the following secondary antibodies: IRDye^®^ 680RD Goat anti-Mouse IgG and IRDye^®^ 800CW Goat anti-Rabbit IgG (diluted 1:10000 in TBS-T, LI-COR, Lincoln, NE). Bound antibodies were visualized via the Odyssey Infrared Imaging System and Odyssey software (LI-COR).

### Prostate Cancer Xenograft Model

Studies utilized male, severe combined immunodeficiency (SCID) beige mice from NCI Frederick Animal Production Area. DU-145 cells were cultured in maintenance media and harvested at 80% confluency. Cells were washed with Dulbecco's phosphate-buffered saline and resuspended at 5 million cells in 100 µL dPBS before injecting subcutaneously into the right flank of each mouse. Mice were monitored, and tumors were measured twice weekly with a caliper. Following resection, tumor volume (mm^3^) was calculated using the formula *V = (W^2^×L)/2.* Whole blood was collected via cardiac puncture and immediately transferred to collection tubes containing K3 EDTA (SAI Infusion Technologies, Lake Villa, IL). Tubes were gently inverted several times and then centrifuged for 15 minutes at 1,500 x g and 4 °C to separate plasma. Tissue and plasma samples were flash-frozen and stored at -80 °C. All animal experiments were approved by NCI Animal Care and Use Committee (ACUC) and followed NCI ACUC guidelines.

### EV RNA Isolation from Culture Medium and Mouse Plasma

Human prostate cancer cells were grown to confluence, washed with Dulbecco's phosphate-buffered saline, and cultivated for two days in serum-free medium. Before EV RNA isolation, culture medium and plasma samples were filtered with a 0.8 µM Millex-AA filter (Millipore). EV RNA was isolated from 25 mL of culture medium and 100-300 µL of mouse plasma using the exoRNeasy Maxi or exoRNeasy Midi Kit (QIAGEN), respectively, per the manufacturer's protocol. MaXtract High-Density tubes (QIAGEN) were used for the solvent extraction phase of this protocol to help maximize RNA recovery and mitigate the potential for contamination.

### Total RNA Isolation from Mouse Tumor

Tumor tissue was thawed on ice, and ~30 mg sections were shaved off using a scalpel. These sections were placed in a bead homogenizer tube with 600 µL RLT buffer (QIAGEN) supplemented with β-Mercaptoethanol (1% v/v) and homogenized for 3 minutes. An equal volume of 70% ethanol (600 µL) was added to the tube and mixed gently to precipitate RNA. The mixture was then transferred to the RNeasy Mini spin column (QIAGEN), and RNA was extracted according to the RNeasy Mini Kit protocol (QIAGEN).

### qPCR detection of SLCO1B3 and ABCC3 in PCa cell line exosomes

Total exosomal RNA was extracted from the supernatant of prostate cancer cell lines (22Rv1, LnCaP, Du145, PC3) using an exoRNeasy kit (QIAGEN). EV RNA concentration was determined using a NanoDrop spectrophotometer (Molecular Devices, Sunnyvale, CA). RNA quality was assessed using a 2100 Bioanalyzer with the RNA 6000 Pico Kit (Agilent Technologies, Santa Clara, CA). The purified EV RNA, cellular and tumor RNA samples were synthesized into cDNA using SuperScript III First-Strand Synthesis System (Invitrogen) using TaqMan quantitative PCR (RT-qPCR) assays as previously described ^30^. Human glyceraldehyde 3-phosphate dehydrogenase (*GAPDH*) or beta-actin (*β-ACTIN*) mRNA was used as an internal control. qPCR products were combined with DNA Gel Loading Dye (Thermo Scientific™) and run on a TBE Gel (Invitrogen). DNA was visualized via the Odyssey Infrared Imaging System and Odyssey software (LI-COR).

### Droplet Digital PCR detection of plasma-derived EV RNA

To detect *ct-SLCO1B3* and *ABCC3* in xenograft plasma-derived EVs, we designed and optimized a droplet digital PCR (ddPCR) assay (BioRad Laboratories, Hercules, CA). Transcription of DU-145 plasma EV RNA into complementary DNA was optimized for *ct-SLCO1B3* and *ABCC3*. We followed the standard first-strand cDNA synthesis protocol with SuperScript™ III Reverse Transcriptase (ThermoFisher Scientific, Waltham, MA) for *ct-SLCO1B3* detection and SuperScript™ IV Reverse Transcriptase for ABCC3 analysis. Primers and probes for *ct-SLCO1B3* were designed using Primer3 software (ThermoFisher Scientific) and developed with fluorescent labels (FAM) by BioRad. *ct-SLCO1B3* Forward Primer: GCCACGTTACTGAATCTACATGTTG; *ct-SLCO1B3* Reverse Primer: CCACCTAGTGCTTTAGCAATATAGCT; Probe: CAGGGCTGCCAAGAACATCTGCTAG. We acquired the commercially available HEX primers and probes for *ABCC3* from BioRad (AssayID: dHsaCPE5049615). We used the absolute quantification capabilities of the QX200 Droplet Generator (BioRad) and followed the manufacturer's protocol. Total reaction volumes of 20 μL were prepared in individual wells of a 96-well semi-skirted PCR plate containing plasma-derived cDNA (or no template control), FAM and HEX primers/probes, DEPC treated water, and 2x ddPCR Supermix for Probes (BioRad). The plasma-derived cDNA was diluted with DEPC water as needed. Reactions were run on the QX200 digital droplet PCR system (BioRad) conducted at the CCR Genomics Core at the National Cancer Institute. Data analysis and rendering were done using QuantaSoft (BioRad).

## Results and Discussion

**Figure 1:**
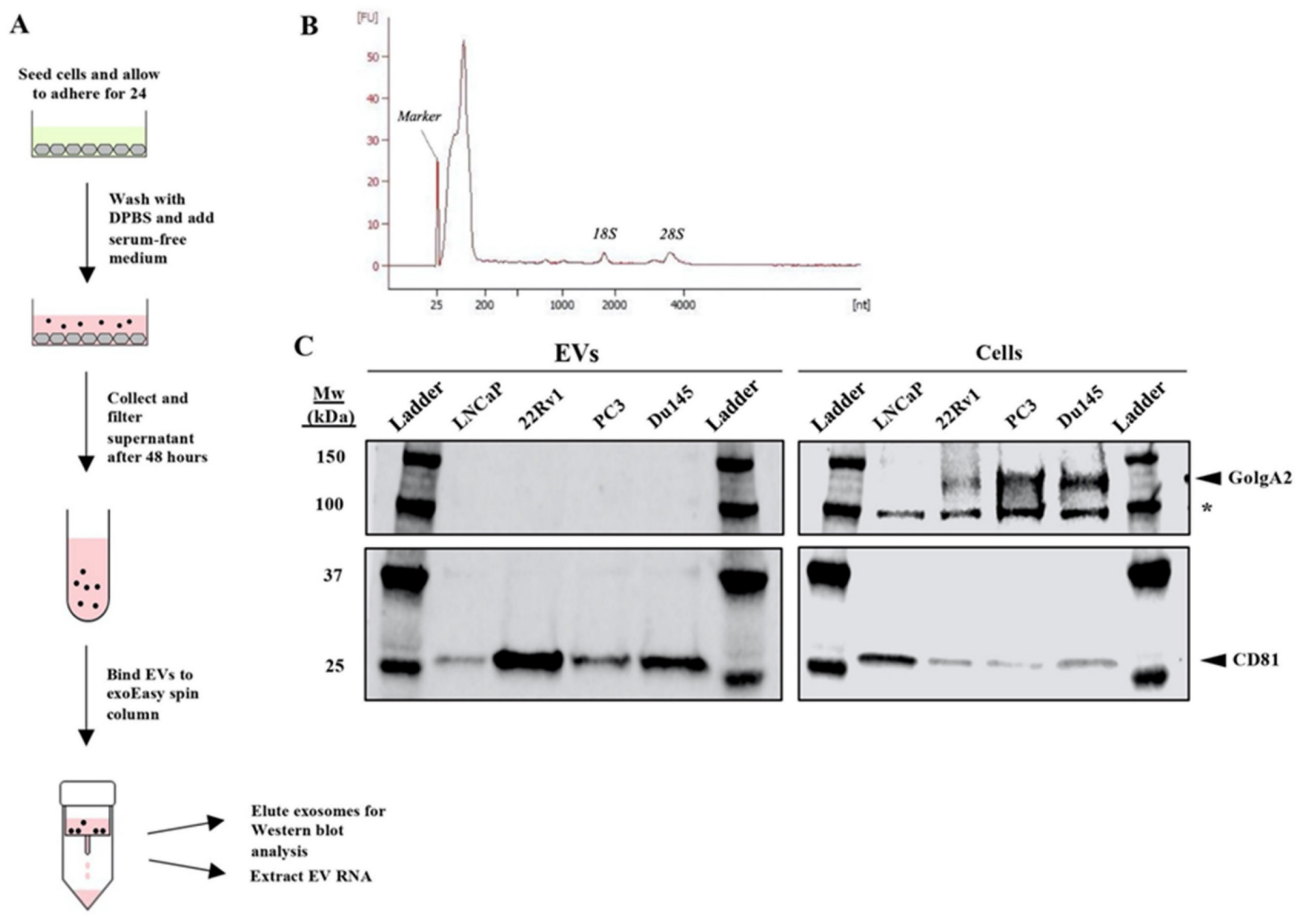
** Purification of exosomes and other EVs with total exosomal RNA isolation from cell culture supernatant using the QIAGEN exoEasy and exoRNeasy protocols.** (A) Experimental diagram of extraction protocol. Each PCa cell line was allowed to adhere for 24 hours, washed once with DPBS, and then cultured in serum-free medium before isolation of EVs followed by EV RNA extractions. (B) A representative electropherogram was produced using the Agilent 2100 Bioanalyzer to confirm that intact EV RNA was successfully extracted from all samples. RNA samples were input as appropriate dilutions to achieve a total RNA concentration of 0.2 - 5 ng/μL per the RNA 6000 Pico Kit guide. (C) Western analysis demonstrated that EVs were both successfully extracted and did not contain cellular carryover, as the exosome-specific marker CD81 (25 kDa) was detectable in each EV extraction and the Golgi protein GolgA2 (130 kDa) only in the whole cell lysate. The* asterisk* denotes a non-specific band. Equivalent amounts of protein were loaded in each lane.

EVs are commonly isolated by ultracentrifugation; however, this study employed the QIAGEN exoEasy and exoRNeasy kits to isolate all EVs and their contents. Similar to ultracentrifugation, the exoRNeasy kit isolates vesicles that are 50-200 nm in size 9. This size range suggests that the extracted EV population is limited to exosomes (40-100 nm) and proteasomes (50-500 nm), both of which have been identified previously as PCa-derived EVs 14. Compared to ultra-centrifugation, the exoRNeasy kit offers a faster, more accessible, and more standardized ap-proach to EV isolation and extracts high-quality EV RNA of equal or better yield 9 (**Figure 1A**).

We first determined the purity of the exoRNeasy kit by examining extracted EVs from four PCa cell lines: LNCaP, 22Rv1, PC3, and DU-145. During this study, all extracted EV RNA quality was assessed using the Agilent 2100 Bioanalyzer (**Figure 1B**). Western blot analysis confirmed the successful extraction of exosomes by confirming the presence of CD81, an exosome-specific protein, in the extracted EV protein of each PCa cell line (**Figure 1C**). The blot also demonstrated that there was no cellular carryover in the EV extraction, as GolgA2, a cellular Golgi protein, was present in the lysates of 22Rv1, PC3, and DU-145 cells but not in extracted EVs (**Figure 1C**). Thus, all analysis of the EV extractions should solely reflect EV content. Analysis of LNCaP EV purity was inconclusive as GolgA2 was undetectable in both the EV extractions and cell lysates (**Figure 1C**).

**Figure 2:**
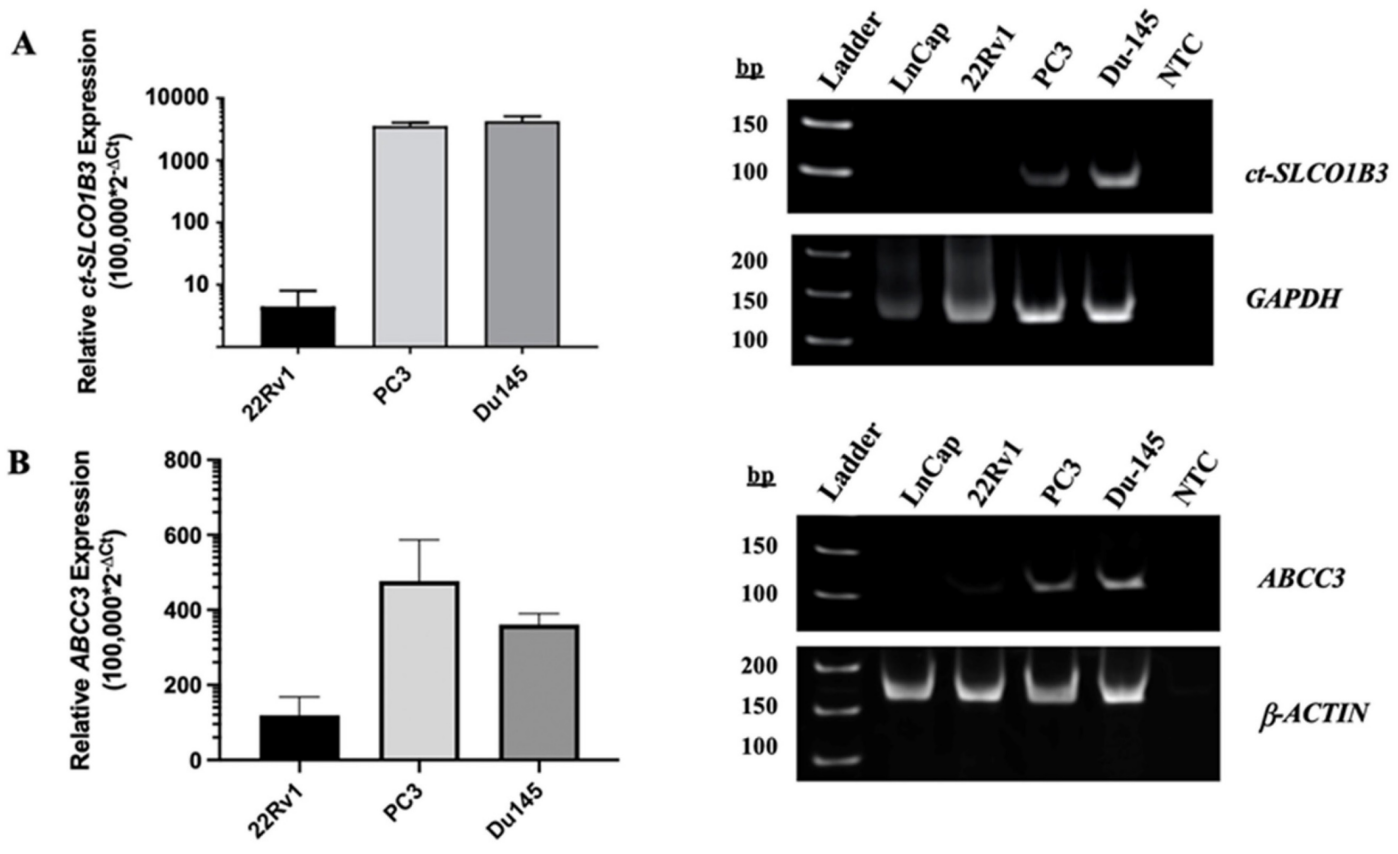
***ct-SLCO1B3* and *ABCC3* are detectable *in vitro* in CRPC cell line-derived EVs.** Total EV RNA was extracted from the cell culture supernatant of prostate cancer cell lines, and (A) *ct-SLCO1B3* and (B) *ABCC3* were quantified via qPCR analysis. Both transcripts were detectable at higher levels in the EV RNA of the CRPC cell lines, PC3 and DU-145, compared to the androgen receptor-positive cell line, 22Rv1. *ct-SLCO1B3* was not detected in LNCaP cells. The qPCR products were analyzed by agarose gel electrophoresis. Human *GAPDH* or *β-ACTIN* mRNA was used as an internal control. All experiments include ≥3 biological and ≥3 technical replicates. NTC refers to non-template control.

To determine whether *ct-SLCO1B3* and *ABCC3* can be detected in prostate cancer EVs, EV RNA was extracted from the supernatant of four PCa cell lines using the method/kit described above. Through qPCR analysis, the EV RNA of each PCa cell line was examined for both *ct-SLCO1B3* and *lt-SLCO1B3* expression. While the *ct-SLCO1B3* (**Figure 2A**) and *ABCC3* (**Figure 2B**) transcripts had high relative gene expression (RGE= 100,000*2^-ΔCt^) in the EV RNA of PC3 (mean RGE=3550) and DU-145 (mean RGE=4255) cells, it was only detectable at low levels in the EV RNA of 22Rv1s (mean RGE=1.3) and undetectable in that of LNCaPs (data not shown). For LNCaPs, there was only one instance in which the *ct-SLCO1B3* transcript was detectable by qPCR in EV RNA (data not shown); however, unlike the other PCa cell lines examined, the transcript was not detectable upon gel analysis (**Figure 2A**). Additionally, *lt-SLCO1B3* was undetectable by qPCR analysis in EV RNA of all the examined PCa cell lines (data not shown). These results demonstrate the presence of *ct-SLCO1B3* and *ABCC3* in PCa EVs and indicate considerably higher expression in the EV RNA of CRPC (AR null) cell lines compared to AR-expressing PCa cell lines, a trend consistent with intracellular patterns of expression ^30^.

**Figure 3:**
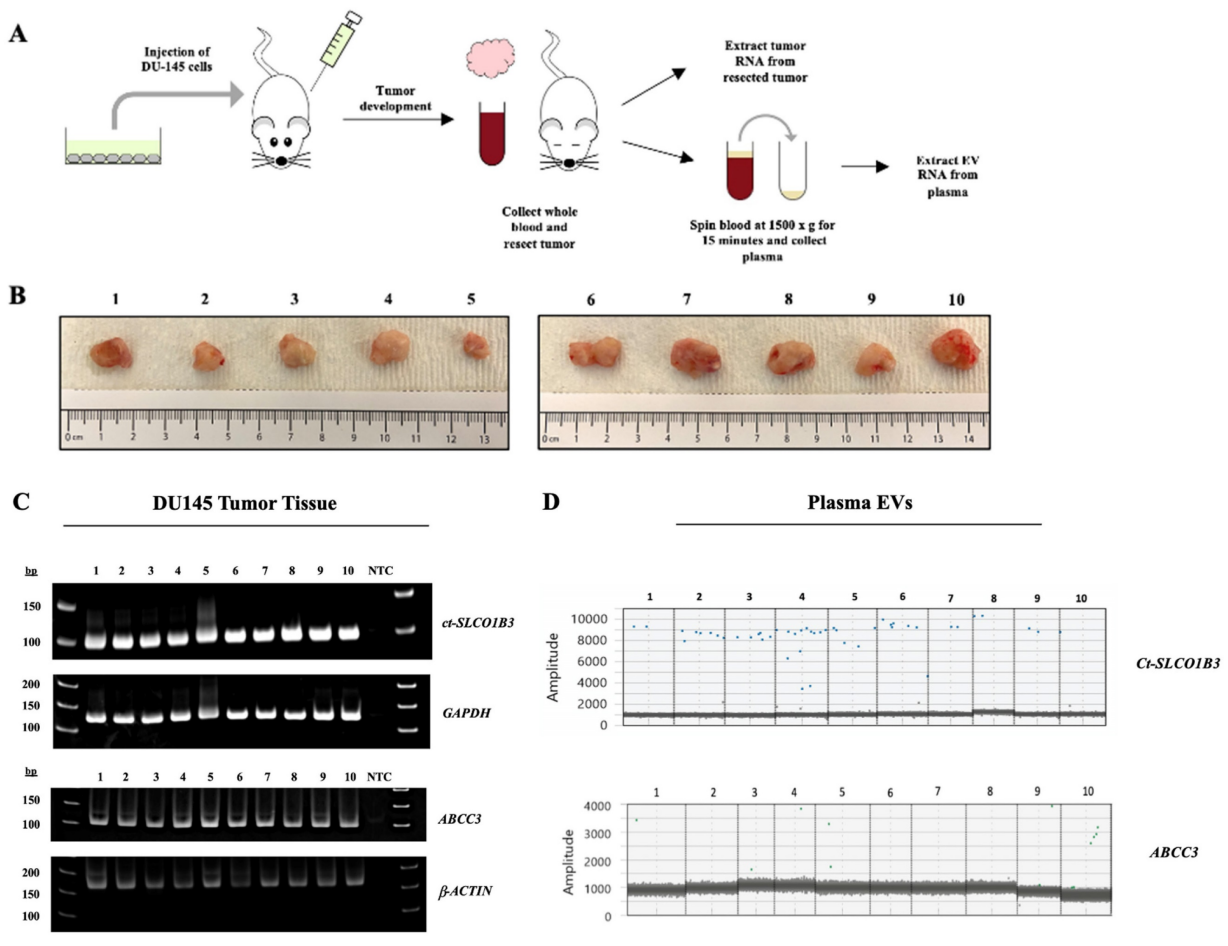
***ct-SLCO1B3* and *ABCC3* are detectable intratumorally and in plasma-derived EVs from the prostate cancer DU-145 xenograft model.** (A) The human prostate cancer xenograft mouse model (using male SCID mice) was developed using DU-145 cells (*n*=10 mice per study). Mice were injected subcutaneously into their right flank with 5x10^6^ DU-145 cells. (B) Representative images of isolated tumors from DU-145 xenografted animals. (C) Total RNA was extracted from each tumor, and qPCR analysis was performed to examine for intratumoral *ct-SLCO1B3* and *ABCC3* mRNA expression. Human *GAPDH* or *β-ACTIN* mRNA was used as an internal control. (D) ddPCR analysis of EV RNA isolated from plasma of each mouse for *ct-SLCO1B3* and *ABCC3* detection with representative 1D amplitude plots shown. NTC refers to non-template control.

We next investigated whether exosomal *ct-SLCO1B3* can be detected *in vivo* using the DU-145 prostate cancer xenograft model. DU-145 cells had a high level of *ct-SLCO1B3* and* ABCC3* expression *in vitro* 27 and in EV-derived RNA. Total RNA and EV RNA were isolated from tumors and plasma, respectively, from male SCID mice bearing DU-145 xenograft tumors (*n*=10, **Figure 3A**). Resected specimens varied in size, with tumor volumes ranging from 150 to 850 mm^3^ (**Figure 3B**). Using qPCR analysis, we detected robust intratumoral expression of Ct-SLCO1B3 transcripts in all extracted tumors (**Figure 3C**). *Ct-SLCO1B3* transcripts were also detectable in all EV RNA extracted from plasma samples via ddPCR analysis (**Figure 3D**). Because the entire plasma volume of EV RNA was utilized to detect *ct-SLCO1B3*, a second DU-145 prostate cancer xenograft study was conducted (*n*=10 animals) to produce more plasma-derived EV RNA to investigate *ABCC3 expression.* An abundance of intratumoral *ABCC3* was detected through a qPCR analysis (**Figure 3C**). Finally, we detected the *ABCC3* transcript in 6 of ten plasma-derived EV RNA samples using ddPCR (**Figure 3D**). There was no correlation between tumor size and abundance of *ct-SLCO1B3* or *ABCC3* transcripts in plasma-derived EVs. Overall, the DU-145 xenograft model demonstrates that PCa tumors expressing *ct-SLCO1B3* and *ABCC3* also secrete EV RNAs expressing both gene transcripts, which can be readily isolated from plasma. Future studies will determine whether *ct-SLCO1B3* and *ABCC3* are detectable in the plasma EVs of CRPC patients.

The presence of *ct-SLCO1B3* in CRPC-derived EVs suggests it may play an essential role in prostate cancer progression. Our laboratory has recently demonstrated the regulation of *SLCO1B3* expression by *hsa-miR-579-3p* 28. Downregulation of hss-miR-579 has also been observed under hypoxic conditions in PCa-derived exosomes 33, and whether expression of EV-derived *ct-SLCO1B3* is similarly miRNA-mediated, specifically by *hsa-miR-579*, remains to be determined. Additionally, miRNAs seem to play a role in EV biogenesis itself. Expression of miR-26a has been shown to suppress EV secretion, and its expression is lower in PCa tissue than in normal tissue 34.

The ATP-binding cassette family members have been well characterized as important membrane transporters of substrates ranging from drugs to metabolic products and steroids 35. Overexpression of *ABCC3* has been reported in several aggressive disease forms, such as pancreatic ductal adenocarcinoma, non-small cell lung cancer, and hepatocellular carcinoma, and is associated with poor prognosis and resistance to treatments 32, 36, 37. Expression of ABCC3 along the human intestine can be regulated by miRNAs 38. ABCC3 was found to be differentially regulated between recurrent and non-recurrent disease among prostate cancer patient samples 39. As such, between EV biogenesis and post-transcriptional regulation, miRNAs appear to play a pivotal role in the expression of *ct-SLCO1B3* and *ABCC3* in EVs, and further investigation into these mechanisms could provide a deeper understanding of the role of exosomal *ct-SLCO1B3* and *ABCC3* in CRPC disease progression.

Exosomes can mediate intercellular communication. Specific exosomal contents have an established association with neuroendocrine transdifferentiation in CRPC patients and can potentially serve as effective diagnostic markers of advanced-stage disease 40. Interestingly, *ct-SLCO1B3* has been shown to be upregulated in non-small cell lung cancer (NSCLC), where it functioned to alter the expression of genes associated with the epithelial-mesenchymal transition (EMT) 41. This genetic reprogramming is a broadly observed phenomenon in cancer biology. There are documented functional connections between EMT and neuroendocrine transdifferentiation 42. Given this association, further work investigating the role of *ct-SLCO1B3* in promoting more aggressive cancer migratory behavior, as well as its putative function in the EMT in prostate cancer, is warranted.

Overall, *ct-SLCO1B3* and *ABCC3* represent promising novel EV-derived RNA biomarkers for PCa progression. A deeper understanding of their expression and regulation in PCa EVs will help clarify the role of *ct-SLCO1B3* and *ABCC3* in CRPC.

## Abbreviations

ABCC3: ATP Binding Cassette Subfamily Member C
ADT: androgen deprivation therapy
CRPC: castration-resistant prostate cancer
CSPC: castration-sensitive prostate cancer
*ct-SLCO1B3*: cancer-type solute carrier organic anion transporter family 1B3
EV: extracellular vesicle
*lt-SLCO1B3*: liver-type solute carrier organic anion transporter family 1B3
PCa: prostate cancer
PSA: prostate-specific antigen

## References

[1] Siegel RL, Miller KD, Wagle NS, Jemal A (2023). Cancer statistics, 2023. CA Cancer J Clin.

[2] Tannock IF, de Wit R, Berry WR, Horti J, Pluzanska A, Chi KN (2004). Docetaxel plus prednisone or mitoxantrone plus prednisone for advanced prostate cancer. N Engl J Med.

[3] Scher HI, Halabi S, Tannock I, Morris M, Sternberg CN, Carducci MA (2008). Design and end points of clinical trials for patients with progressive prostate cancer and castrate levels of testosterone: recommendations of the Prostate Cancer Clinical Trials Working Group. J Clin Oncol.

[4] Ionescu F, Zhang J, Wang L (2022). Clinical Applications of Liquid Biopsy in Prostate Cancer: From Screening to Predictive Biomarker. Cancers (Basel).

[5] Matuszczak M, Schalken JA, Salagierski M (2021). Prostate Cancer Liquid Biopsy Biomarkers' Clinical Utility in Diagnosis and Prognosis. Cancers (Basel).

[6] Morio H, Sun Y, Harada M, Ide H, Shimozato O, Zhou X (2018). Cancer-Type OATP1B3 mRNA in Extracellular Vesicles as a Promising Candidate for a Serum-Based Colorectal Cancer Biomarker. Biol Pharm Bull.

[7] Sun Y, Woess K, Kienzl M, Leb-Reichl VM, Feinle A, Wimmer M (2018). Extracellular Vesicles as Biomarkers for the Detection of a Tumor Marker Gene in Epidermolysis Bullosa-Associated Squamous Cell Carcinoma. J Invest Dermatol.

[8] Becker A, Thakur BK, Weiss JM, Kim HS, Peinado H, Lyden D (2016). Extracellular Vesicles in Cancer: Cell-to-Cell Mediators of Metastasis. Cancer Cell.

[9] Enderle D, Spiel A, Coticchia CM, Berghoff E, Mueller R, Schlumpberger M (2015). Characterization of RNA from Exosomes and Other Extracellular Vesicles Isolated by a Novel Spin Column-Based Method. PLoS One.

[10] Scott E, Munkley J (2019). Glycans as Biomarkers in Prostate Cancer. Int J Mol Sci.

[11] Soung YH, Ford S, Zhang V, Chung J (2017). Exosomes in Cancer Diagnostics. Cancers (Basel).

[12] Brzozowski JS, Jankowski H, Bond DR, McCague SB, Munro BR, Predebon MJ (2018). Lipidomic profiling of extracellular vesicles derived from prostate and prostate cancer cell lines. Lipids Health Dis.

[13] Clos-Garcia M, Loizaga-Iriarte A, Zuniga-Garcia P, Sanchez-Mosquera P, Rosa Cortazar A, Gonzalez E (2018). Metabolic alterations in urine extracellular vesicles are associated to prostate cancer pathogenesis and progression. J Extracell Vesicles.

[14] Vlaeminck-Guillem V (2018). Extracellular Vesicles in Prostate Cancer Carcinogenesis, Diagnosis, and Management. Front Oncol.

[15] Ramirez-Garrastacho M, Bajo-Santos C, Line A, Martens-Uzunova ES, de la Fuente JM, Moros M (2022). Extracellular vesicles as a source of prostate cancer biomarkers in liquid biopsies: a decade of research. Br J Cancer.

[16] Diao Y, Zhu B, Ding T, Li R, Li J, Yang L (2023). Tumor-derived extracellular vesicle nucleic acids as promising diagnostic biomarkers for prostate cancer. Front Oncol.

[17] Fujita K, Nonomura N (2018). Urinary biomarkers of prostate cancer. Int J Urol.

[18] Kretschmer A, Tutrone R, Alter J, Berg E, Fischer C, Kumar S (2022). Pre-diagnosis urine exosomal RNA (ExoDx EPI score) is associated with post-prostatectomy pathology outcome. World J Urol.

[19] Wang L, Wang J, Yin X, Guan X, Li Y, Xin C, Liu J (2022). GIPC2 interacts with Fzd7 to promote prostate cancer metastasis by activating WNT signaling. Oncogene.

[20] Zavridou M, Strati A, Bournakis E, Smilkou S, Tserpeli V, Lianidou E (2021). Prognostic Significance of Gene Expression and DNA Methylation Markers in Circulating Tumor Cells and Paired Plasma Derived Exosomes in Metastatic Castration Resistant Prostate Cancer. Cancers (Basel).

[21] Bryant RJ, Pawlowski T, Catto JW, Marsden G, Vessella RL, Rhees B (2012). Changes in circulating microRNA levels associated with prostate cancer. Br J Cancer.

[22] Guo T, Wang Y, Jia J, Mao X, Stankiewicz E, Scandura G (2020). The Identification of Plasma Exosomal miR-423-3p as a Potential Predictive Biomarker for Prostate Cancer Castration-Resistance Development by Plasma Exosomal miRNA Sequencing. Front Cell Dev Biol.

[23] Antonarakis ES, Lu C, Wang H, Luber B, Nakazawa M, Roeser JC (2014). AR-V7 and resistance to enzalutamide and abiraterone in prostate cancer. N Engl J Med.

[24] Del Re M, Biasco E, Crucitta S, Derosa L, Rofi E, Orlandini C (2017). The Detection of Androgen Receptor Splice Variant 7 in Plasma-derived Exosomal RNA Strongly Predicts Resistance to Hormonal Therapy in Metastatic Prostate Cancer Patients. Eur Urol.

[25] Hamada A, Sissung T, Price DK, Danesi R, Chau CH, Sharifi N (2008). Effect of SLCO1B3 haplotype on testosterone transport and clinical outcome in caucasian patients with androgen-independent prostatic cancer. Clin Cancer Res.

[26] Sharifi N, Hamada A, Sissung T, Danesi R, Venzon D, Baum C (2008). A polymorphism in a transporter of testosterone is a determinant of androgen independence in prostate cancer. BJU Int.

[27] Sissung TM, Ley AM, Strope JD, McCrea EM, Beedie S, Peer CJ (2017). Differential Expression of OATP1B3 Mediates Unconjugated Testosterone Influx. Mol Cancer Res.

[28] Barbier RH, McCrea EM, Lee KY, Strope JD, Risdon EN, Price DK (2021). Abiraterone induces SLCO1B3 expression in prostate cancer via microRNA-579-3p. Sci Rep.

[29] Yang M, Xie W, Mostaghel E, Nakabayashi M, Werner L, Sun T (2011). SLCO2B1 and SLCO1B3 may determine time to progression for patients receiving androgen deprivation therapy for prostate cancer. J Clin Oncol.

[30] Alam K, Farasyn T, Ding K, Yue W (2018). Characterization of Liver- and Cancer-type-Organic Anion Transporting Polypeptide (OATP) 1B3 Messenger RNA Expression in Normal and Cancerous Human Tissues. Drug Metab Lett.

[31] Thakkar N, Kim K, Jang ER, Han S, Kim K, Kim D (2013). A cancer-specific variant of the SLCO1B3 gene encodes a novel human organic anion transporting polypeptide 1B3 (OATP1B3) localized mainly in the cytoplasm of colon and pancreatic cancer cells. Mol Pharm.

[32] Ramirez-Cosmes A, Reyes-Jimenez E, Zertuche-Martinez C, Hernandez-Hernandez CA, Garcia-Roman R, Romero-Diaz RI (2021). The implications of ABCC3 in cancer drug resistance: can we use it as a therapeutic target?. Am J Cancer Res.

[33] Panigrahi GK, Ramteke A, Birks D, Abouzeid Ali HE, Venkataraman S, Agarwal C (2018). Exosomal microRNA profiling to identify hypoxia-related biomarkers in prostate cancer. Oncotarget.

[34] Urabe F, Kosaka N, Sawa Y, Yamamoto Y, Ito K, Yamamoto T (2020). miR-26a regulates extracellular vesicle secretion from prostate cancer cells via targeting SHC4, PFDN4, and CHORDC1. Sci Adv.

[35] Klaassen CD, Aleksunes LM (2010). Xenobiotic, bile acid, and cholesterol transporters: function and regulation. Pharmacol Rev.

[36] Adamska A, Ferro R, Lattanzio R, Capone E, Domenichini A, Damiani V (2019). ABCC3 is a novel target for the treatment of pancreatic cancer. Adv Biol Regul.

[37] Zhao Y, Lu H, Yan A, Yang Y, Meng Q, Sun L (2013). ABCC3 as a marker for multidrug resistance in non-small cell lung cancer. Sci Rep.

[38] Bruckmueller H, Martin P, Kahler M, Haenisch S, Ostrowski M, Drozdzik M (2017). Clinically Relevant Multidrug Transporters Are Regulated by microRNAs along the Human Intestine. Mol Pharm.

[39] Karatas OF, Guzel E, Duz MB, Ittmann M, Ozen M (2016). The role of ATP-binding cassette transporter genes in the progression of prostate cancer. Prostate.

[40] Bhagirath D, Liston M, Akoto T, Lui B, Bensing BA, Sharma A, Saini S (2021). Novel, non-invasive markers for detecting therapy induced neuroendocrine differentiation in castration-resistant prostate cancer patients. Sci Rep.

[41] Hase H, Aoki M, Matsumoto K, Nakai S, Nagata T, Takeda A (2021). Cancer type-SLCO1B3 promotes epithelial-mesenchymal transition resulting in the tumour progression of non-small cell lung cancer. Oncol Rep.

[42] Dicken H, Hensley PJ, Kyprianou N (2019). Prostate tumor neuroendocrine differentiation via EMT: The road less traveled. Asian J Urol.

